# Symbiotic compatibility between rice cultivars and arbuscular mycorrhizal fungi genotypes affects rice growth and mycorrhiza-induced resistance

**DOI:** 10.3389/fpls.2023.1278990

**Published:** 2023-10-24

**Authors:** Ludivine Guigard, Lea Jobert, Nicolas Busset, Lionel Moulin, Pierre Czernic

**Affiliations:** PHIM Plant Health Institute, Univ Montpellier, IRD, CIRAD, INRAE, Institut Agro, Montpellier, France

**Keywords:** *Oryza sativa*, plant-fungi interactions, biological control, *Xanthomonas oryzae*, symbiotic association, biotic stress

## Abstract

**Introduction:**

Arbuscular mycorrhizal fungi (AMF) belong to the Glomeromycota clade and can form root symbioses with 80% of Angiosperms, including crops species such as wheat, maize and rice. By increasing nutrient availability, uptake and soil anchoring of plants, AMF can improve plant’s growth and tolerance to abiotic stresses. AMF can also reduce symptoms and pathogen load on infected plants, both locally and systemically, through a phenomenon called mycorrhiza induced resistance (MIR). There is scarce information on rice mycorrhization, despite the high potential of this symbiosis in a context of sustainable water management in rice production systems.

**Methods:**

We studied the symbiotic compatibility (global mycorrhization & arbuscules intensity) and MIR phenotypes between six rice cultivars from two subspecies (*indica*: IR64 & Phka Rumduol; *japonica*: Nipponbare, Kitaake, Azucena & Zhonghua 11) and three AMF genotypes (*Funneliformis mosseae* FR140 (FM), *Rhizophagus irregularis* DAOM197198 (RIR) & *R. intraradices* FR121 (RIN)). The impact of mycorrhization on rice growth and defence response to *Xanthomonas oryzae* pv *oryzae* (Xoo) infection was recorded via both phenotypic indexes and rice marker gene expression studies.

**Results:**

All three AMF genotypes colonise the roots of all rice varieties, with clear differences in efficiency depending on the combination under study (from 27% to 84% for Phka Rumduol-RIN and Nipponbare-RIR combinations, respectively). Mycorrhization significantly (α=0.05) induced negative to beneficial effects on rice growth (impact on dry weight ranging from -21% to 227% on Azucena-FM and Kitaake-RIN combinations, respectively), and neutral to beneficial effects on the extent of Xoo symptoms on leaves (except for Azucena-RIN combination which showed a 68% increase of chlorosis). *R. irregularis* DAOM197198 was the most compatible AMF partner of rice, with high root colonisation intensity (84% of Nipponbare’s roots hyphal colonisation), beneficial effects on rice growth (dry weight +28% (IR64) to +178% (Kitaake)) and decrease of Xoo-induced symptoms (-6% (Nipponbare) to -27% (IR64)). Transcriptomic analyses by RT-qPCR on leaves of two rice cultivars contrasting in their association with AMF show two different patterns of response on several physiological marker genes.

**Discussion:**

Overall, the symbiotic compatibility between rice cultivars and AMF demonstrates adequate colonization, effectively restricting the nutrient starvation response and mitigating symptoms of phytopathogenic infection.

## Introduction

In recent years, there has been a growing interest in the naturally occurring interactions between plants and the inhabitants of their root microbiome. It has been widely reported that this cohort of microorganisms plays a role in the growth of their host and in its tolerance to biotic and abiotic stresses (Berendsen et al., 2012; Schlaeppi and Bulgarelli, 2015; Vannier et al., 2019; de la Fuente Cantó et al., 2020). These include arbuscular mycorrhizal fungi (AMF), which form a mutualistic association with the roots of various crops such as wheat, maize or rice. (Paszkowski and Boller, 2002; Suzuki et al., 2015; Fiorilli et al., 2018).

The establishment of this symbiosis is mediated by a molecular dialogue between the partners, via the exudation of strigolactones by the plant and the recognition of fungal Myc factors (lipochitooligosaccharides or short-chain chitin oligomers) (Mbodj et al., 2018; Ho-Plágaro and García-Garrido, 2022). When in contact with the root, the hyphae changes into an adhesive structure named hyphopodia, enabling the access to the internal root cortex. It spreads *via* intercellular spaces and colonise cortical cells with highly branched intracellular structures named arbuscules, preferential sites of exchange with their host (Gutjahr et al., 2008; Gutjahr et al., 2009; Jung et al., 2012; Liu et al., 2022). Their number and functioning in a plant root system is recognised as a marker of symbiotic compatibility (Montero et al., 2019).

During these exchanges, plants retribute up to 30% of their produced photosynthates to the fungi in the form of sugars and lipids (Jung et al., 2012; Sugiura et al., 2020). In return, mycorrhizal fungi provide a multitude of beneficial effects. The mere presence of the fungi within cortical root cells can enhance shoot and root biomass, especially stimulating the lateral roots formation and development in wheat, rice or maize (Oláh et al., 2005; Gutjahr et al., 2009; Chiu et al., 2018; Fiorilli et al., 2018). Its hyphal network enables it to cover a large area of soil, mineralising and recovering essential and/or poorly bioavailable nutrients or water for the development of its host (Begum et al., 2019; Kadam et al., 2020). Particularly, phosphorus is a key nutrient in AMF symbiosis with plants (Smith et al., 2011). Its bioavailability (or lack thereof due to complexation with soil particulates) affects the recruitment and functioning of the fungal association with its host (Breuillin et al., 2010; Jiang et al., 2021). Mycorrhization impacts inorganic phosphate (Pi) responsive genes expression in multiple plant species, such as rice or wheat, suppressing for instance the Pi starvation response typically occurring in low Pi soils (Yang et al., 2012; Fiorilli et al., 2018; Campo and San Segundo, 2020). In addition to improving the mineral nutrition of the plant, the AMF symbiosis can help its host tolerate a wide range of stresses. It can increase its host’s tolerance to a variety of abiotic stresses, from drought to excessive temperature, or reduce root uptake of heavy metals (de Andrade et al., 2015; Begum et al., 2019; Chen et al., 2019). AMF symbioses also enhance their host’s tolerance to pathogen pressure at two complementary levels. Fungal root colonisation protects the host both by competing with soil pathogens for its photosynthates and colonisation sites and by triggering a local defence response (accumulation of callose, ROS, phenols and R proteins) (Schouteden et al., 2015; Gupta et al., 2017; Dowarah et al., 2021). By modulating phytohormonal pathways such as jasmonate, salicylic acid and ethylene, AMF primes its host’s defence responses in the shoot via a mechanism called mycorrhiza-induced resistance (MIR) (Jung et al., 2012; Gupta et al., 2017; Fiorilli et al., 2018; Nishad et al., 2020). This MIR results in reduced foliar symptoms and control of pathogen development on a variety of plants and shoot pathogens (Liu et al., 2007; Fiorilli et al., 2018; Kadam et al., 2020). Due to their ability to improve soil fertility and plant health, AMF have great potential as plant bioinoculants in the field.

This potential is as promising as AMF are generally known to have low host specificity, capable to induce growth of a multitude of different crops (Van Geel et al., 2016). However, recent meta-analyses and studies highlighted that there exists different symbiotic compatibilities between AMF species and crop cultivars since different associations results in different phenotypic observations (Pérez-de-Luque et al., 2017; Campos et al., 2018; Silva et al., 2018). A meta-analysis on 115 studies showed that some specific associations between AMF genera and plant host families are more efficient for crop growth promotion, such as the *Poacees* family with the AMF genera *Funneliformis* and *Rhizophagus* (Van Geel et al., 2016). Crop responsiveness to mycorrhization is indeed plant genotype-dependent, as well as AMF species-dependent and is positively linked with AMF colonisation (Lehmann et al., 2012). This meta-analysis explained that the relationship between crop mycorrhizal response and AMF colonisation wasn’t significant for wheat or barley, while recent studies have underlined differences in symbiotic compatibility between rice genotypes, both in terms of AMF colonisation and its effect on rice growth (Suzuki et al., 2015; Diedhiou et al., 2016; Davidson et al., 2019).

Rice typical watering mode is flooding, but it has major drawbacks: monopolising a third of the world’s freshwater, high levels of methane production, soil polluting streaming of chemical inputs and negative impact on AMF development (Redeker et al., 2000; Chandel et al., 2002; Saito et al., 2018; Chialva et al., 2020). As a substitute to constant flooding, Alternate Wetting and Drying (AWD) rice management practices have been developed, reducing water use by up to 30% and methane emissions by 48% without reducing yield (Richards and Sander, 2014; LaHue et al., 2016). Rice varieties that are AMF-responsive should therefore be selected in fields that are being converted from flooded to AWD rice systems.

Within these paddy fields, initial studies showed little or no colonisation of different rice varieties under flooded conditions (Lumini et al., 2011; Vallino et al., 2014), but recent ones have reported AMF colonisation in experimental and farmers’ fields around the world, especially in rainfed lowland systems (Chialva et al., 2020; Sarkodee-Addo et al., 2020; Barro et al., 2022). There are few studies on the natural occurrence of AMF communities and their diversity in rice paddy fields (Wang et al., 2015; Bernaola et al., 2018a; Zhang et al., 2020; Wang et al., 2021). AMF communities rely on the site and the irrigation mode and *Glomerales, Archaeosporales* and *Diversisporales* are generally the predominant orders (Lumini et al., 2011; Chialva et al., 2020; Barro et al., 2022). Members from the *Glomeraceae, Claroideoglomeraceae* and *Paraglomeraceae* families have been found in paddy fields in China and Ghana (Wang et al., 2015; Sarkodee-Addo et al., 2020), including the well-studied *Rhizophagus irregularis* and *Funneliformis mosseae* species. Another analysis on fragrant black rice in Indian fields identified *R. intraradices* and *F. mosseae* in both field and rice root samples (Surendirakumar et al., 2021). Their global distribution in various fields, long-term storage ability, and ability to form symbiosis with a wide range of plant hosts make them excellent models for studying AMF symbiosis (Berruti et al., 2016).

Under greenhouse conditions, there is evidence that rice mycorrhization can improve plant biomass, yield and tolerance to multiple abiotic and biotic stresses (Gutjahr et al., 2009; Campos-Soriano et al., 2012; Li et al., 2016; Campo et al., 2020). These beneficial effects depend on rice developmental stage, variety and AMF genotype (Suzuki et al., 2015; Sisaphaithong et al., 2017; Wang et al., 2021). Surprisingly, studies on the global effect of AMF symbiosis on both rice’s growth and defence responses are scarce or limited to a few combinations of rice cultivars and AMF species. Studies by Campos-Soriano & San-Segundo showed that the mycorrhization of a single *japonica* variety, Senia, by the AMF model *R. intraradices* enhances its biomass and resistance to *Pyricularia (P.) oryzae*, both locally and systemically (Campos-Soriano et al., 2010; Campos-Soriano et al., 2012). Two AMF species, *R. irregularis* and *F. mosseae*, inoculated on 12 *japonica* rice varieties showed contrasted effects on rice growth, Pi content in leaves, and resistance to *P. oryzae* infection (Campo et al., 2020). A multi-AMF species inoculant on another two tropical *japonica* varieties also increases their growth but at the same time their susceptibility to insect attacks and *Rhizoctonia solani* infection (Bernaola et al., 2018b). Finally, a large study by Suzuki et al., 2015 showed a range of impact (from improvement to deterioration) of *F. mosseae*’s inoculation on the biomass of 64 rice genotypes.

In order to develop AMF bioinoculants for rice production under AWD conditions, it is necessary to deepen our understanding of the symbiotic compatibility between rice and AMFs. This will assess which combinations are beneficial, negligible, or detrimental to plant growth and responses to environmental stresses.

In this study, the symbiotic compatibility between six varieties of *japonica* and *indica* rice and three AMF genotypes, known to interact with rice, was characterised. Model rice varieties as well as varieties with potential for AWD programs were targeted. We analysed the colonisation rate and intensity as well as the functioning of the interaction between rice and mycorrhizal fungi. We then assessed how AMF inoculation impacts rice’s growth and defence responses to a pathogenic infection by *Xanthomonas oryzae* pv. *oryzae (*Xoo). We used a combination of phenotypic and rice gene expression studies to uncover promising compatible associations. How phenotypic responses of rice to AMF symbiosis can be linked to systemic changes in marker gene expression (ranging from growth, phytohormonal balances to defence response) was investigated in the leaves of two rice model cultivars contrasting in AMF establishment and responses.

## Materials and methods

### Plant and fungal material

Six *Oryza sativa* cultivars and three AMF genotypes belonging to three different species were selected and their characteristics are listed in **Table 1**. Two *indica* (IR64 and Phka Rumduol) and four *japonica* (Azucena, Kitaake, Nipponbare and Zhonghua 11) subspecies were selected. Seeds were obtained from IRRI and propagated at IRD except for Phka Rumduol which was provided by CIRAD.

**Table 1:** Rice cultivars and AMF genotypes used in this study.

Plant/Fungal Material	Characteristics	Reference
*Oryza sativa* subsp. *japonica*		
Nipponbare	*japonica* reference, high-quality sequenced genome, AMF-responsive, drought sensitive	Gutjahr et al., 2008; Matsumoto et al., 2016; Degenkolbe et al., 2009
Kitaake	model for rice transformation, short cycle, not light sensitive, AMF-responsive, drought tolerant to a certain extent	Jain et al., 2019; Mubarok et al., 2019; Shi et al., 2021
Azucena	short cycle, not light sensitive, sensitive to phytoparasitic nematodes, not yet tested on AMF symbiosis drought tolerant	Masson et al., 2022; Ghorbanzadeh et al., 2023
Zhonghua 11	short cycle, not light sensitive, resistant to phytoparasitic nematodes, widely used in China for T-DNA mutant sources, AMF-responsive drought sensitive	Phan et al., 2018; Huang et al., 2020; Masson et al., 2022; Nguyen et al., 2022; Xiao et al., 2009
*Oryza sativa* subsp. *indica*		
IR64	*indica* reference, high yield quality, sensitive to nematodes, AMF-responsive, drought sensitive	Suzuki et al., 2015; Mackill and Khush, 2018; Phan et al., 2018; Ghorbanzadeh et al., 2023
Phka Rumduol	jasmine premium rice, highly cultivated in Cambodia, not yet tested on AMF symbiosis drought sensitive	Masson et al., 2022; Zhao et al., 2016
AMF genotypes		
*Funneliformis mosseae* FR140	Colonise a large variety of hosts including rice, worldwide presence in fields, induce MIR in rice against *Pyricularia* (P.) *oryzae* and in wheat against *Xanthomonas oryzae.* Colonise a large variety of hosts including rice, worldwide presence in fields, induce MIR in rice against *P. oryzae*	Vos et al., 2012; Suzuki et al., 2015; Berruti et al., 2016; Fiorilli et al., 2018; Campo et al., 2020.
*Rhizophagus intraradices* FR121		Gutjahr et al., 2008; Campos-Soriano et al., 2012; Berruti et al., 2016.
*Rhizophagus irregularis* DAOM197198	Colonise a large variety of hosts including rice, long-term storage, worldwide presence in fields, sequenced genome, induce MIR in rice against *P. oryzae.*	Stockinger et al., 2009; Tisserant et al., 2013; Berruti et al., 2016; Campo et al., 2020.

*Funneliformis mosseae* FR140 (FM), *Rhizophagus intraradices* FR121 (RIN) and *Rhizophagus irregularis* DAOM 197198 (RIR) were purchased from MycAgro Lab (Technopôle Agro-Environnement, Bretenière, France) in the form of individual granular inoculums (100 spores/g).

### Plant growth conditions

Rice seeds were dehusked and surface-sterilised by immersion in 70% ethanol for 3 min, then in 3.8% sodium hypochlorite supplemented with 1% Tween 20 under agitation (180 rpm) for 30 min. Seeds were rinsed three times with sterile water, three times with 2% filtered sodium thiosulfate and three more times with sterile water. They were incubated overnight at 28°C in sterile water in the dark and then germinated on sterile-soaked sand for three days at 28°C. To ensure the absence of contaminants, 100 µL of the last rinse water and imbibition water were plated on tryptic soy agar (Sigma-Aldrich) Petri dishes. Four homogeneous rice seedlings were then transferred to anti-coiling pots (Comptoir Vert, France) filled with 150 mL of clay beads (6-18 mm, Terres & Traditions, France) and 450 mL of sterile inert substrate composed of 70% of sand, 20% sieved perlite and 10% of vermiculite (Campo & San Segundo, 2020). This substrate was inoculated with either the AMF granular inoculum, or the granular inoculum without fungal spores (control) at a volume of 5% per pot.

Rice plants were grown for 2.5 months in a growth chamber (12 h day/night, 28°C day, 26°C night, 75% humidity). The substrate was moistened for one week and then watered three times a week with a Hoagland solution (Hoagland and Arnon, 1938) reduced in phosphate (2.5 mM Ca(NO_3_)_2_, 2.5 mM KNO_3_, 1 mM MgSO_4_, 0.25 mM (NH_4_)_2_SO_4_, 25 µM KH_2_PO_4_ and trace elements, complete recipe in **Supplementary Table 1**), with the watering volume gradually increased according to the cultivar growth.

### Rice growth phenotyping

Maximum height, shoot and root fresh weights were measured for each plant (n = 20 per condition). Shoot dry weight was also measured after drying at 40°C for one week at 48 h (n = 20 par condition). Roots were stored in 70% ethanol at 4°C until mycorrhizal quantification (n= 5 pools of 4 root systems).

### Mycorrhizal quantification

The root systems of four plants from the same pot were washed in tap water, placed in 70% ethanol and stored at 4°C until analysis. Fungal structures were stained using a blue ink-based protocol modified from Cao et al., 2013. Roots were heated to 80°C for 45 min in 10% KOH. They were rinsed three times with ultrapure water (MilliQ) and stained with a staining solution consisting of 5% blue ink (Waterman “Bleu Sérénité”) in 5% acetic acid at room temperature for 10 min. They were then rinsed three times with ultrapure water and fixed in 5% acetic acid overnight.

Five replicates of 20 to 25 fragments from coronary roots were mounted between slide and glass and the mycorrhizal index (global mycorrhization and arbuscular intensity) were assessed as in Trouvelot et al., 1986, on a Axiozoom Zeiss microscope.

### Biocontrol assays against *Xanthomonas oryzae* pv *oryzae*

Infection of *Xanthomonas oryzae* pv. *oryzae* PXO99 (Xoo) was carried out on 50-days old rice plants by leaf clipping as described in Niño-Liu et al., 2005. The extent of chlorosis and necrosis was assessed 14 days post inoculation (dpi) on each leaf (n = 18). Mock inoculations were made with sterile water to assess that the sole clipping of the leaf does not induce any disease symptoms.

### RNA extraction and quantitative PCR of rice gene expression

RNA was extracted from leaf samples collected during leaf-clipping (before infection, at 50 days post germination). They were ground to a fine powder using a TissueLyser II (Retsch) at 30 Hz, for 15 s twice. Each biological replicate consisted of two (Nipponbare) to three (IR64) leaf samples from the same pot, with four biological replicates for each condition. Total RNA was extracted using TriReagent (Sigma) and a DNAse (QIAgen) treatment was added in the protocol before purification with the RNA Clean & Concentrator kit (Zymo), according to the manufacturer’s instructions. The quantity and quality of total RNA was assessed using a NanoDrop 1000 spectrophotometer (ThermoFisher). Approximately 360 ng of total RNA from each biological replicate was used for retrotranscription into cDNA using SuperScript III Reverse Transcriptase (Thermo Fisher Scientific). cDNAs were diluted 5-fold and RT-qPCRs were performed using the Takyon™ Low ROX SYBR 2X MasterMix blue dTTP (Eurogentec) on a LightCycler 480 qPCR system (Roche). Plate preparation was automated using the epMotion 5070 pipetting robot (Eppendorf). Four independent biological replicates were analysed for each condition, each one analysed in triplicate. Relative gene expression is calculated by comparing each sample to the standard’s (number of cycles of EF1a, ΔCt ), and then to the control group (FM, RIN & RIR vs. CT, ΔΔCt ). The fold change is calculated with $2^{-mean\Delta \Delta Ct}$ and the logFC represents the relative expression of each marker gene as indicated in (Pfaffl, 2001). The list of marker genes, their function and the primers used in this study are listed in **Table 2**.

**Table 2:** Rice marker genes used in RT-qPCR expression studies.

Gene name	Annotation RAPDB	Forward primer (5’-3’)	Reverse primer (5’-3’)	References
Reference gene				
***OsEF-1A***	Os03g0177400 - Rice elongation factor 1A	GAAGTCTCATCCTACCTGAAGAAG	GTCAAGAGCCTCAAGCAAGG	Petitot et al., 2017
Defence response				
***OsWRKY30***	Os08g0499300 - WRKY transcription factor, Disease resistance against X. Oryzae, Drought tolerance	ATGGCTGTCTGTCAGAGAGGATG	CAGTGGTAGGAGAAGGTTGTGC	Ryu et al., 2006
***OsMPK10***	Os01g0629900 - Similar to Blast and wounding induced mitogen-activated protein kinase	TCAACTCCAATTCCTGCCAAG	AACAACTCTTCCTGGTCTTGC	Nguyễn et al., 2014
***OsPAL4***	Os02g0627100 - Phenylalanine ammonia-lyase, Broad spectrum disease resistance	CCTCGCCATCGCTGCCATC	GCCGTTGTTGTAGAAGTCGTTCAC	Petitot et al., 2017
***OsPR5***	Os12g0628600 - Similar to Thaumatin-like pathogenesis-related protein 3 precursor	CGCTGCCCCGACGCTTAC	ACGACTTGGTAGTTGCTGTTGC	Delteil et al., 2012
***OsTGAP1***	Os04g0637000 - TGA-type bZIP Transcription Factor, Regulation of diterpenoid phytoalexin production, Defence response	ATGGCCAGTGAAGGATGAAG	CTCTTGTGCCCACATCAGAA	Okada et al., 2009
***OsDXS3***	Os07g0190000 - Similar to 1-deoxy-D-xylulose 5-phosphate synthase 2 precursor	TGTTCTTGCCAGACAGGTAC	GTCGGCTGATGTGTATATGC	Valette et al., 2020
Hormone (SA)				
***OsNPR1***	Os01g0194300 - Ankyrin-repeat protein, Herbivore-induced defence response, Blast disease resistance	AGAAGTCATTGCCTCCAG	ACATCGTCAGAGTCAAGG	Kumari et al., 2016
***OsWRKY45***	Os05g0322900 - WRKY transcription factor, Benzothiadiazole (BTH)-inducible blast resistance	CGGGCAGAAGGAGATCCAAAACT	GCCGATGTAGGTGACCCTGTAGC	Shimono et al., 2012
Hormone (JA)				
***OsJAMyb***	Os11g0684000 - JA-dependent myb transcription factor	TAGGGGTTCAAAGAGGACCA	TCCTCAGTGCAATTCTGGAG	Yokotani et al., 2013
***OsJAZ6***	Os03g0402800 - TIFY family protein, JASMONATE-ZIM domain (JAZ) protein, JA signalling, Regulation of spikelet development	TTGATGACTTCCAGCTGAGAA	GCGCTGTGGAGGAACTCTTG	Lu et al., 2016
***OsLOX4***	Os03g0700400 - Lipoxygenase-3, Generation of stale flavour	TGGTGGAGCAGATCTACGTG	ATCGCCTTGATCGAGTAGCC	Nguyen et al., 2022
Hormone (ET)				
***OsACS1***	Os03g0727600 - ACC synthase, Ethylene biosynthesis	GATGGTCTCGGATGATCACA	GTCGGGGGAAAACTGAAAAT	Petitot et al., 2017
Nutrient homeostasis				
***OsNIA1***	Os02g0770800 - NADH/NADPH-dependent nitrate reductase	AAGGTGTCTTGTGCTGGATGGC	AGCTTGTCGAGTTCGTCCTTGC	Tang et al., 2012
***OsIRO2***	Os01g0952800 - Iron-related bHLH transcription factor 2, Tolerance to Fe deficiency, Regulation of Fe uptake from soil, Fe translocation to grain during seed maturation	ACGAGCTCTACTCCTCCCTC	CTTCTGCAGCTCGGGTATGT	Ogo et al., 2006
***OsMGD2***	Os08g0299400 - Monogalactosyl diacylglycerol (MGDG) synthase, Adaptation to Pi deficiency, Phosphate utilisation and acquisition	AGACAGGTTGCCAGATGGTT	CTGGAGCTTGTGGATGTCCT	Hasegawa et al., 2010
***OsPAP23***	Os08g0280100 - Purple acid phosphatase 23	GACTCTGGTTGGTTGTGTGC	GCATCAGCGTGTTCATGGAA	Secco et al., 2013
***OsSPX3***	Os10g0392600 - SPX domain-containing protein, Negative regulation of phosphate signalling, Pi homeostasis	CAGTCCATCCGATCCGATCC	TCTCTCAATGACTCGTTTCGT	Secco et al., 2013
Development				
***OsXTH17***	Os08g0237000 - Xyloglucan endotransglucosylases/hydrolase, Cell wall modification processes during rice growth and development	GCCGACTTCCACACCTACAA	GCCAGGTCGTCGTACTTCTT	Lin et al., 2019
***OsYABBY6***	Os12g0621100 - Similar to Filamentous flower-like yabby protein	TTCGTCGTCTTCCAAGCTCA	ACCCTTTGCCTCTTCTCTGG	Jiang et al., 2015

### Statistical analyses

Statistical analyses were performed with Rstudio (version 2022.2.0.443) and R (version 4.1.3) softwares using the packages “readxl”, “tidyverse”, “ggplot2”, “rstatix”, “ggpubr”, “multicompView”, “car”, “pcr”, “pheatmap”. As phenotypic data of mycorrhization and arbuscular intensity indexes fulfilled normality (Shapiro test, p>0.05) and homoscedasticity (Levene test, p>0.05) hypotheses, a two-way ANOVA was used to test the significance of rice cultivar and AMF genotype effects on these parameters, and one-way ANOVA with Tukey test were used for pairwise group comparisons. For rice growth and biocontrol traits, these data did not fulfil the normality and homoscedasticity hypotheses, thus a non-parametric ANOVA with Kruskal-Wallis test (α=0.05) followed by pairwise comparisons with Wilcoxon tests (α=0.05) were used for mean group comparisons of measured phenotypic traits.

For the analysis of the expression of rice marker genes, the “pcr” package was used. Linear regression was used to assess statistical differences between AMF inoculation and marker gene expression. To visualise the relative expression of each marker gene as a function of rice cultivars and AMF inoculation, a heatmap of LogFC was made with the “pheatmap” package.

## Results

### Mycorrhizal colonisation and arbuscular content in AMF-rice *japonica* and *indica* rice cultivars

**Figure 1:**
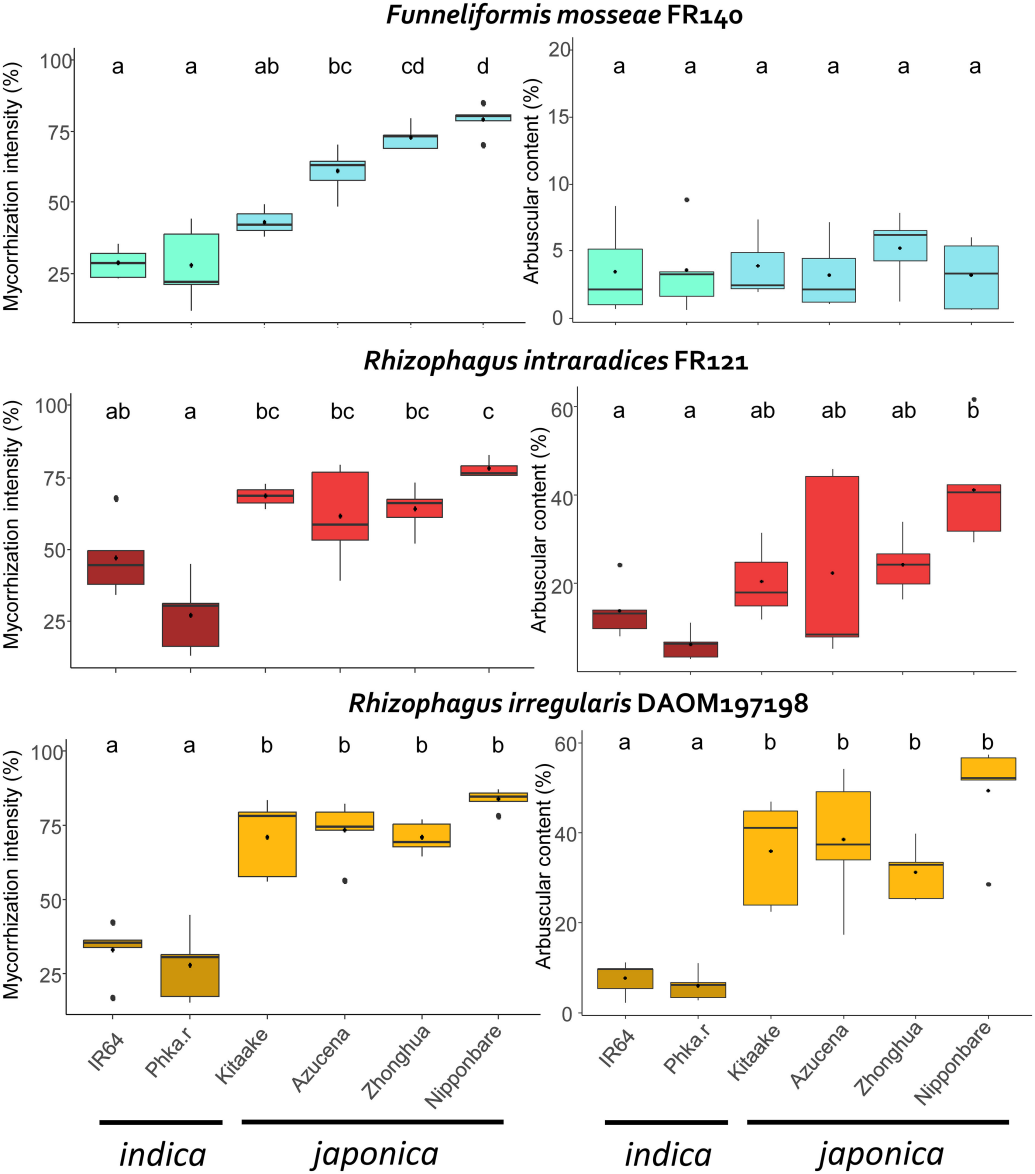
Mycorrhization index of each AMF genotype according to rice cultivar. Left: global mycorrhization intensity (%), right: arbuscular content (%). Blue: *F. mosseae*, red: *R. intraradices*, yellow: *R. irregularis.* Turquoise, dark red and dark yellow: *indica* rice. Light blue, red and yellow: *japonica* rice. Letters above boxplots indicate statistical groups according to the results of the ANOVA and Tukey tests (p value< 0.05). Phka.r: Phka Rumduol; Zhonghua: Zhonghua 11.

To assess the symbiotic compatibility between the three AMF genotypes (RIN, RIR, FM) and the six rice cultivars (listed in **Table 1**), we analysed the mycorrhization rates in the 18 combinations. We focused on the global fungal colonisation rate and the percentage of visible arbuscules in the mycorrhizal roots (see Material & Methods). The use of mycorrhizal index of global mycorrhization and arbuscular intensity allowed us to quantify the interaction between AMF and rice. The mean of each mycorrhizal index for each combination are presented in **Figure 1** and in **Supplementary Table 2**.

We tested whether rice cultivar and AMF genotype have an impact on rice’s global mycorrhization and arbuscular intensity index, with a two-way ANOVA test. Both indexes are statistically significantly affected by either rice cultivar or AMF genotype (Two-Way ANOVA, F = 66.91593, p< 2.10^-6^; F = 4.9315, p< 0.01).

**Figure 2:**
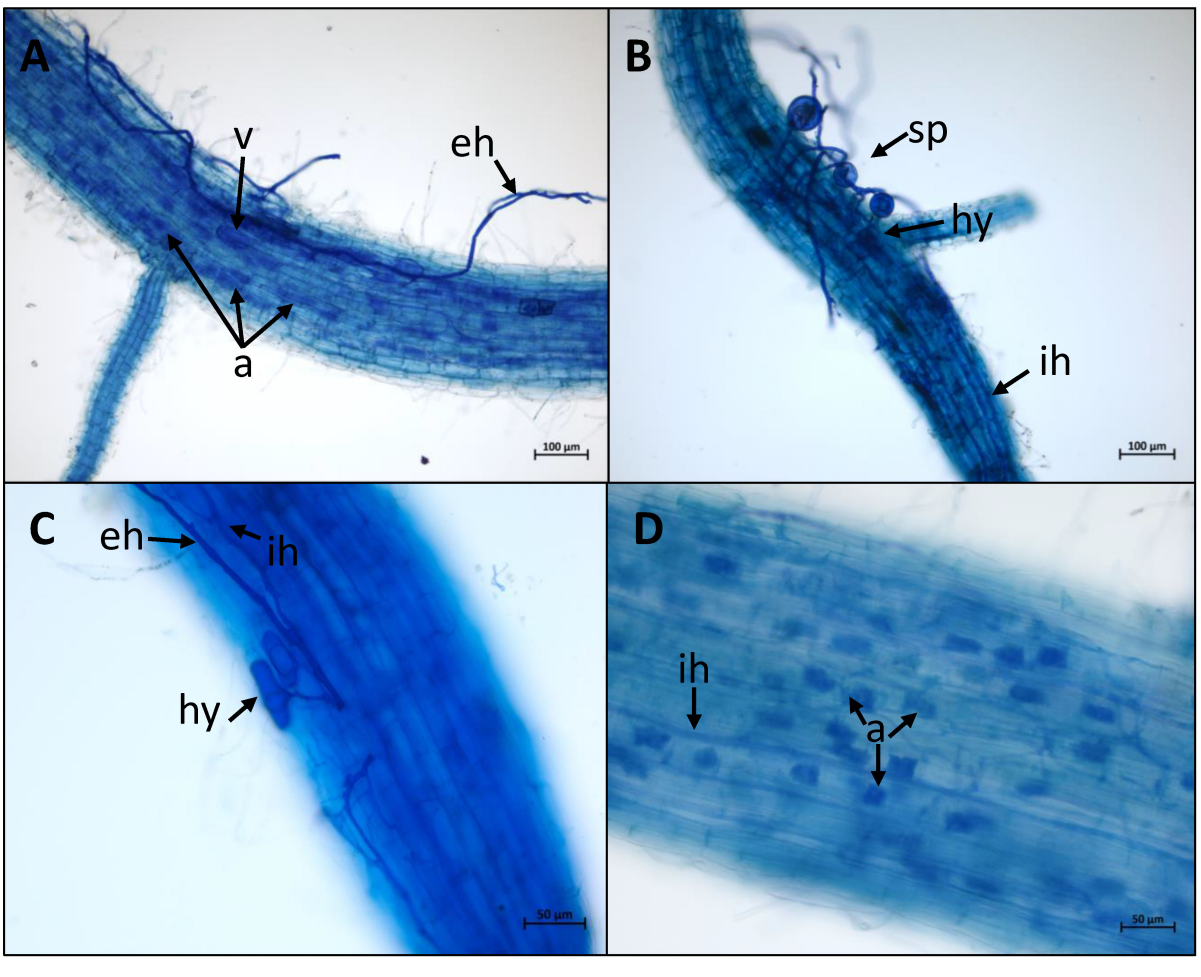
AMF colonisation in rice roots. Plants were stained using the ink-acetic acid method. External fungal organs: external hyphae (eh), spores (sp). Symbiotic fungal organs: intercellular hyphae (ih); hyphopodia (hy); vesicule (v); arbuscules (a). **(A)** = Colonisation of Nipponbare roots by *R. intraradices* FR121. **(B)** = Colonisation of Nipponbare roots by *F. mosseae* FR140. **(C)** = Colonisation of Azucena roots by *R. irregularis* DAOM197198. **(D)** = Colonisation of Zhonghua 11 roots by *R. intraradices* FR121.

All the rice cultivars tested were root colonised by each AMF genotype (**Figure 1**; **Supplementary Table 2**). Each fungal organ (hyphal structures, spores, vesicles and arbuscules) was clearly visible on each combination as shown in **Figure 2** and **Supplementary Figure 1**. The global intensity of mycorrhization ranged from 27.20% (Phka Rumduol with RIN) to 83.90% (Nipponbare with RIR). Independently of the fungal inoculation, *indica* rice varieties have the less intense symbiotic percentage, ranging from 27.20% (Phka Rumduol - RIN) to 46.8% (IR64 - RIN) (**Supplementary Table 2**). The percentage of mycorrhization of the *japonica* cultivars ranges from 43.20% (Kitaake with FM) to 83.90% (Nipponbare with RIR). Nipponbare is the most intensely mycorrhized cultivar with 79%, 77.90% and 83.90% for FM, RIN and RIR inoculation, respectively (**Supplementary Table 2**).

The arbuscular percentage of the mycorrhizal roots ranged from 3.22% (Azucena with FM) to 49.40% (Nipponbare with RIR) (**Supplementary Table 2**). *Japonica* rice cultivars showed the highest percentage of visible arbuscules in the mycorrhized system compared to *indica* rice, with RIN and (**Figure 1**). On the other hand, each rice genotype in interaction with FM formed almost no arbuscules, with a maximum of 5.24% in Zhonghua 11 (**Supplementary Table 2**).

### Growth response of rice cultivars to AMF colonisation

**Figure 3:**
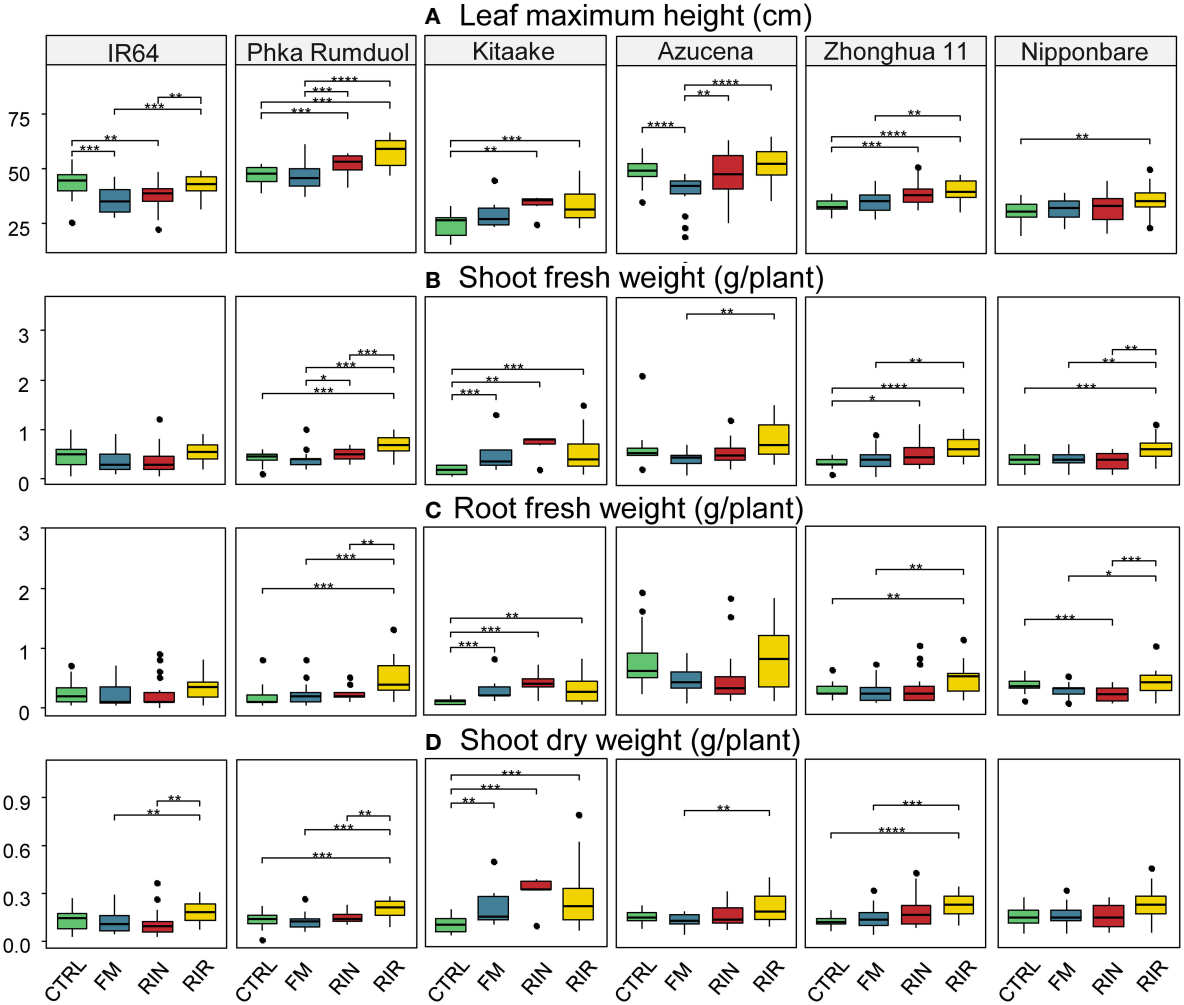
Effect of mycorrhization on phenotypic traits for each combination. **(A)** Maximum leaf height (cm). **(B)** Shoot fresh weight (g/plant). **(C)** Fresh root weight (g/plant). **(D)** Shoot dry weight (g/plant), depending on AMF inoculation. Green box plot: CTRL (no AMF), blue: FM (*F. mosseae* FR140*)*, red: RIN (*R. intraradices* FR121*)*, yellow: RIR (*R. irregularis* DAOM197198). n=20 for each combination except Kitaake-RIN and Kitaake-FM with n=6 and n=8, respectively. *: p value< 0.05. **: p value< 0.01. ***: p value< 0.005. ****: p value<< 0.005 (Wilcoxon test; adjusted pvalue with Bonferroni method).

The phenotypic response of rice to AMF inoculation and symbiosis establishment was assessed by growth measurements. Maximum height, fresh and dry shoot weight and fresh root weight were measured for each combination (n = 20) and are shown as boxplots in **Figure 3**. All the corresponding measured values are listed in **Supplementary Table 2**. Developmental stage of each cultivar (at 10 weeks) is shown in **Supplementary Figure 2**. Plants were still at developmental vegetative stage, except for Kitaake plants which flowered one week before harvest.

The dataset shows that AMF inoculation can result in an increase, decrease or no significant effect on plant growth parameters. We observed a significant decrease in the height of IR64 during the interaction with FM or RIN (-18.81% and -14.12% respectively, **Figure 3A** and **Supplementary Table 2**). The RIR genotype resulted in a significant increase in both root and shoot weights for Phka Rumduol, Kitaake, Zhonghua 11 and Nipponbare. However, this effect was not statistically significant for Azucena. The effect on rice’s dry weight wasn’t as significant as on height or fresh weight, but was still a good proxy for the beneficial effect of AMF on rice growth (**Figure 3**).

Under our growth conditions, we observed different growth rates depending on the rice cultivar. Uninoculated Kitaake was the smallest rice cultivar both in size (24.65 cm) and weight (0.19 g, 0.11 g, 0.09 g on average for fresh shoot, root and dry shoot weight, respectively; **Supplementary Table 2**). Still, mycorrhization of Kitaake induced a clear improvement in growth: the highest dry weight among all combinations being obtained with Kitaake in association with RIN (0.30 g, **Figure 3D**; **Supplementary Table 2**). This combination showed the greatest positive effect on rice’s growth on all variables: 36% taller, 259%, 270% and 221% heavier on biomass of fresh shoots and roots, and dry shoots, respectively (**Supplementary Table 2**).

Globally, FM inoculation affected rice growth non-significantly (Nipponbare, Phka Rumduol, Zhonghua 11) or negatively (Azucena, IR64), in both height and weight (**Figure 3**). The only significant positive interaction was with Kitaake: 20% taller, 163%, 177%, 125% heavier on its fresh shoot & root and dry shoot weights, respectively (**Figure 3**; **Supplementary Table 2**). The effect of RIN inoculation was contrasting: beneficial on Kitaake and Zhonghua 11, negative on IR64 and Phka Rumduol or non-significant on Azucena and Nipponbare (**Figure 3**). RIR was the AMF genotype that induce the most positive effects among all rice cultivars: +35% and +20% leaf height with Kitaake and Phka Rumduol, respectively (**Figure 3A**); +182% and 103% fresh shoot weight with Kitaake and Zhonghua 11, respectively (**Figure 3B**) and +196% and +149% fresh root weight with Kitaake and Phka Rumduol, respectively (**Figure 3C**).

Our results show that the effect of AMF inoculation on rice growth depends on both rice cultivars and AMF genotypes: ranging from negative, neutral to beneficial outcomes across the 18 combinations under study.

### Mycorrhiza-induced resistance

**Figure 4:**
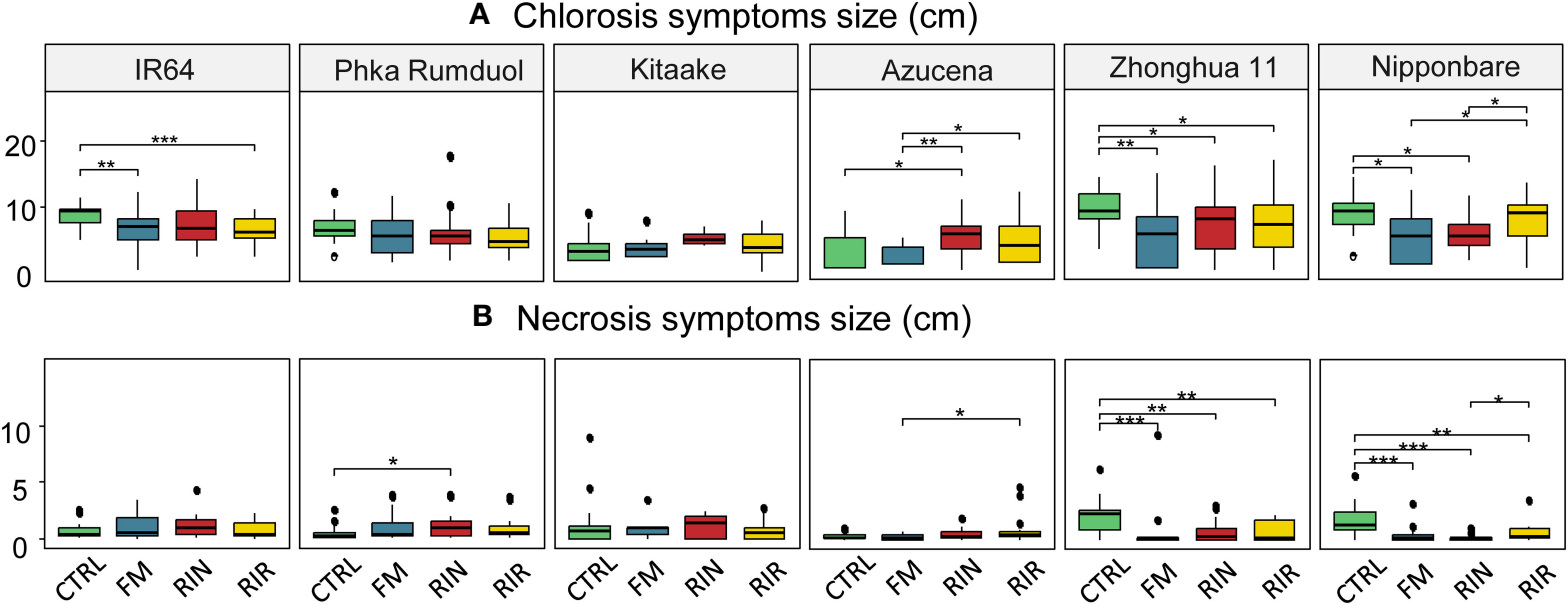
Size of chlorosis **(A)** and necrosis **(B)** symptoms following leaf clipping with Xoo, for each rice cultivar-AMF genotype combination. Green: no AMF, blue: *F*. *mosseae* FR140, red: *R. intraradices* FR121, yellow: *R. irregularis* DOAM197198. n=18 for each combination except Kitaake-RIN and Kitaake-FM with n=5 and n=7, respectively. *: p value< 0.05. **: p value< 0.01. ***: p value< 0.005 (Wilcoxon test).

The potential of each fungal inoculum to induce systemic resistance in the leaves of each rice cultivar during a shoot phytopathogen infection was investigated. Rice plants were infected by leaf-clipping with *Xanthomonas oryzae* pv *oryzae* PXO99 (Xoo) and the extent of chlorosis and necrosis was recorded 14 days later. The results are shown as boxplots in **Figure 4** and all the corresponding measured values are listed in **Supplementary Table 2**.

The effect of AMF inoculation on the chlorosis and necrosis symptoms of rice induced by Xoo differed greatly between combinations. Only two combinations, both with RIN, showed a significant increase in leaf symptoms: Azucena on chlorosis (+69%) and Phka Rumduol on necrosis (+106%). Regarding the bio-protective effects of AMF, chlorosis symptoms were significantly reduced on IR64 in combination with FM (-24%) and RIR (-26%), on Zhonghua 11 in combination with FM (-44%), RIN (-28%) and RIR (-29%), and on Nipponbare with FM (-40%) and RIN (-34%) (**Figure 4** and **Supplementary Table 2**). For necrosis, only Zhonghua 11 and Nipponbare showed significant reductions of symptoms. These reductions in the size of necrosis were observed with the three AMF genotypes: -65%, -66% and -64% for Zhonghua 11 with FM, RIN and RIR respectively; and -78%, -87% and -64% for Nipponbare with FM, RIN and RIR, respectively (**Figure 4** and **Supplementary Table 2**).

### RT-qPCR analysis of growth and immunity molecular marker genes in contrasting rice-AMF combinations

**Figure 5:**
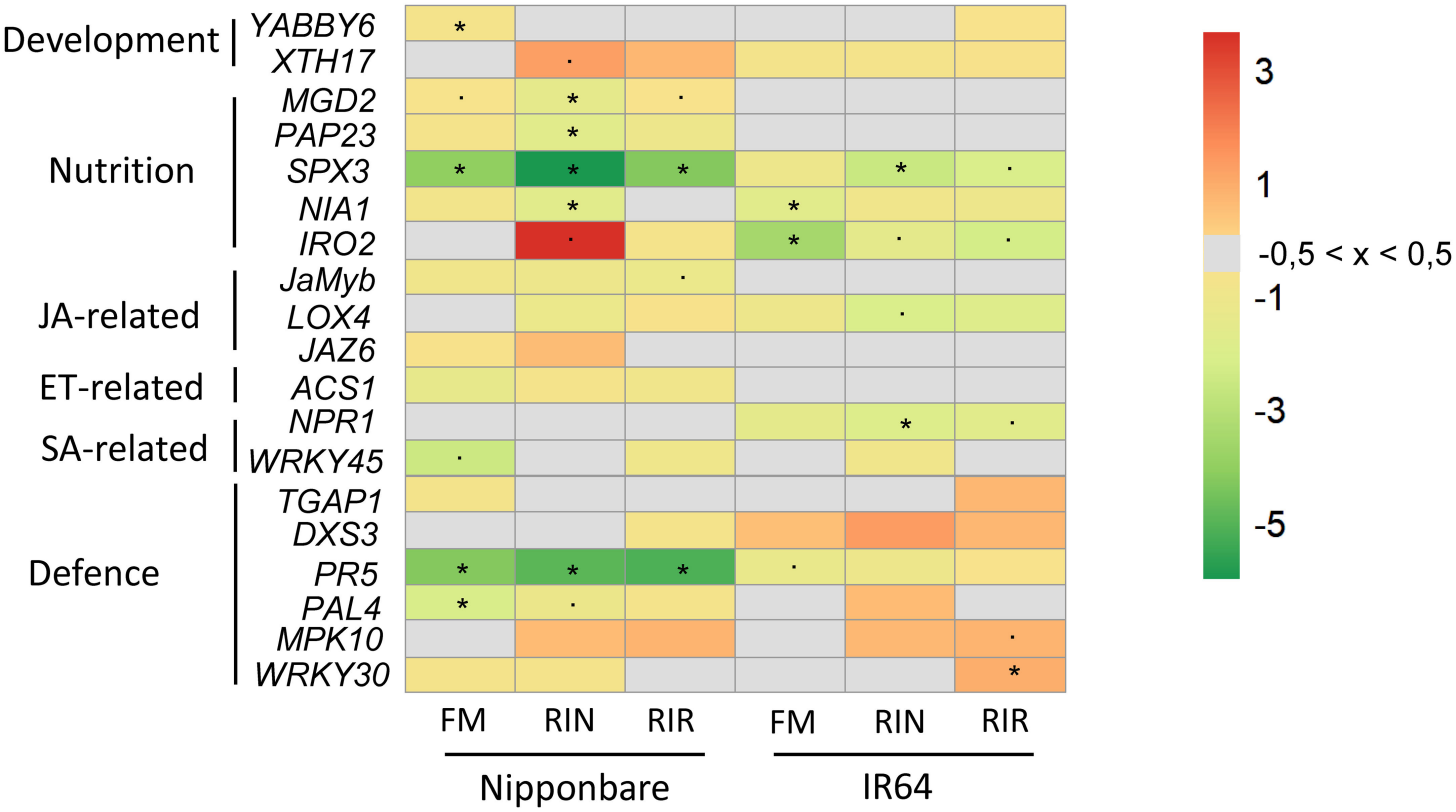
Relative expression of rice marker genes in response to mycorrhization, in Nipponbare and IR64 leaves. Marker genes related to development, nutrition, hormone balance and defence were selected from the literature (listed in **Table 2**). Transcript levels were normalised to that of the reference gene *EF1a*. The log2 fold change values are shown in red (positive), green (negative) and grey (between -0.5 and 0.5) (n = 4). JA= jasmonic acid. ET= ethylene. SA= salicylic acid. “*”= statistically significant (p-value< 0.05). “.”= tendencies (p-value< 0.10).

We observed contrasted patterns of symbiotic compatibility among our AMF-cultivar combinations. In order to link these observed differences with the expression level of leaf marker genes, we selected two rice cultivars with contrasting AMF responses: Nipponbare and IR64. The first is a *japonica* model cultivar and was the most intensely mycorrhized, regardless of the AMF genotype, with the interaction having non-significant to beneficial effects on its growth and tolerance to Xoo infection (**Figures 1**, **3**, **4**). The latter is an *indica* model cultivar, that was significantly less mycorrhized, with non-significant to negative effects of the AMF interaction on its growth, but with beneficial effects on its tolerance to Xoo infection (**Figures 1**, **3**, **4**). We selected 19 markers genes of development, nutrient homeostasis, hormonal balances and defence and their expression was normalised to that of *EF1a* reference gene. The list of marker genes, their function and the primers used in this study are listed in **Table 2**. A summary of the statistical comparison of gene expression for each combination, for both Nipponbare and IR64, is provided in **Supplementary Table 3** and **Supplementary Figures 3**, **4**. Their expression was visualised as a heatmap in **Figure 5**.

The expression of two cellular growth marker genes, *OsYABBY6*, responsible for abaxial-adaxial polarity and whose expression is needed for leaf development (Jiang et al., 2015), and *OsXTH17*, a xyloglucan endotransglucosylase/hydrolases involved in primary cell wall formation (Lin et al., 2019) was recorded. A non-significant induction of *OsXTH17* expression was observed for Nipponbare in interaction with RIN (p= 0.055), while that of *OsYABBY6* is repressed with FM (p=0.009) (**Figure 5**; **Supplementary Table 3**; **Supplementary Figure 3**). In IR64, their expression was not significantly affected independently of the AMF genotype (**Supplementary Figure 4**).

To assess the effect of mycorrhization on mineral homeostasis in leaves, one iron transporter (*OsIRO2*), one nitrate-reductase (*OsNIA1*) and three Pi transporters, marker genes of Pi-starvation response (*OsMGD2*, *OsPAP23* and *OsSPX3*) were selected. The expression of almost all mineral marker genes was significantly reduced in Nipponbare leaves (*OsNIA1*, *OsMGD2*, *OsPAP23* with RIN, *OsSPX3* with either AMF genotype), except for a non-significant strong induction of *OsIRO2* expression when associated with RIN (p= 0,09) (**Figure 5**; **Supplementary Table 3**). In leaves of IR64, the expression of *OsMGD2* and *OsPAP23* was not significantly affected. The expression of the other mineral marker genes was reduced only significantly for *OsSPX3* in RIN-mycorrhized leaves, and for *OsNIA1* and *OsIRO2* in FM-mycorrhized leaves (**Figure 5**; **Supplementary Table 3**; **Supplementary Figures 3**, **4**).

Mycorrhization is known to affect the hormonal balance in mycorrhized plants. Its effect on the expression of jasmonate (JA: *OsLOX4*, a lipoxygenase responsible for the biosynthesis of JA (Nguyen et al., 2022) and *OsJAMyb* & *OsJAZ6*, both responsible for JA signalling (Yokotani et al., 2013; Lu et al., 2016), ethylene (ET: *OsACS1*, 1-aminocyclopropane-1-carboxylate synthase responsible for ethylene biosynthesis) and salicylic acid (SA: *OsNPR1*, mediating SA biosynthesis and responsives genes (Kumari et al., 2016) & *OsWRKY45*, a transcription factor mediating SA signalling) pathways was investigated. Overall, jasmonate- and ethylene-related genes expression was not significantly repressed in Nipponbare and IR64 leaves (**Figure 5**; **Supplementary Table 3**). SA-related genes were not significantly repressed, except for *OsNPR1* in RIN mycorrhized-IR64 leaves (**Figure 5**; **Supplementary Table 3**; **Supplementary Figures 3**, **4**).

The expression of defence-related genes was recorded to assess how mycorrhization affects the defence response in healthy leaves. *OsPR5* is a pathogenesis-related protein, *OsPAL4* is a broad-spectrum disease resistance-related gene and *OsTGAP1* & *OsDXS3* are responsible for phytoalexines production in rice (Okada et al., 2009; Delteil et al., 2012; Petitot et al., 2017; Valette et al., 2020). *OsMPK10* and *OsWRKY30* are responsible for early disease-mediated signalling, the latter also responsive to SA and JA treatments (Ryu et al., 2006; Nguyễn et al., 2014). Globally, defence genes appeared to be more induced in mycorrhized IR64 leaves than in Nipponbare leaves (**Figure 5**; **Supplementary Figures 3**, **4**). In Nipponbare leaves, we observed i) a significant repression of *OsPR5* expression, irrespective of the AMF species, ii) a significant down-regulation of *OsPAL4* expression with FM, iii) a non-significant down-regulation with RIN (**Supplementary Table 3**). In leaves of IR64, phytoalexin biosynthesis-related genes (*OsDXS3* and *OsTGAP1*) were not significantly induced in plant associated with AMF. When mycorrhized with RIR, the expression of *OsWRKY30* is significantly induced but not the one of *OsMPK10* (p-value = 0.03 and 0.07, respectively) (**Figure 5**; **Supplementary Table 3**).

## Discussion

### Symbiotic compatibility between six rice cultivars and three AMF genotypes depends on the studied combination

In this study we have characterised the symbiotic compatibility between multiple rice cultivars from *japonica* and *indica* subspecies in association with three AMF genotypes (*F. mosseae* FR140*, R. intraradices* FR121 and *R. irregularis* DAOM197198). First, we observed that all rice cultivars selected for this study were colonised by each fungal inoculum, their colonisation intensity differing between rice cultivars (Phka Rumduol being the lowest and Nipponbare the highest in terms of global mycorrhization). A similar pattern was also observed at the arbuscular level, with FM developing fewer arbuscules when interacting with rice, compared to the two *Rhizophagus* genotypes. We also observed an effect of AMF colonisation on rice growth that could be either beneficial, neutral, or negative. However, there was no direct relationship between the level of colonisation and the growth phenotype: both *indica* rice cultivars had similar AMF colonisation and arbuscular levels, but the tendency of IR64 to be negatively affected on growth wasn’t observed for Phka Rumduol (**Figures 1**, **3**). The *japonica* cultivars used in this study were generally beneficially affected in their growth (either in height or weight; **Figure 3**). A special case was observed with the cultivar Kitaake. As stated earlier, early mortality and a general lack of growth were observed for this cultivar in our inert substrate. We hypothesised that Kitaake’s growth wasn’t optimal under these drastic conditions. However, plants that have managed to grow were well colonised by the three AMF (43%, 69% and 71% of roots colonised by FM, RIN, and RIR respectively). The highest levels of growth improvement were recorded for this cultivar: 37% taller, 259%, 270% and 221% heavier on biomass of fresh shoots and roots and dry shoots (**Supplementary Table 2**). AMF symbiosis is known to enhance root anchoring and nutrient uptake in poor soils, thereby improving plant health (Paszkowski and Boller, 2002; Gutjahr et al., 2009; Chiu et al., 2018). However, early growth depression following mycorrhization has also been documented in wheat, barley, and soybean (Jacott et al., 2017). Such depression was eventually overcome (or not) in the later life cycle of the crop (Jacott et al., 2017). This growth depression can be explained by the genetic variability of AMF genotypes and their symbiotic effectiveness, but the recurring hypothesis is related to the trade-off between plant photosynthates and soil nutrients recovered by AMF (Jin et al., 2017). Our differences in the phenotypic growth effects of mycorrhization among rice cultivars could be explained by an imbalance between early life stage development and the photosynthetic carbon cost for AMF establishment and function (Jacott et al., 2017; Jin et al., 2017). Our growth conditions were adapted from recent studies on wheat and rice mycorrhization, composed of a mixture of sand, sieved perlite, and vermiculite. It mimics sandy soil and is easy to sterilise. Though, this inert substrate is still poor in essential nutrients, and a modified Hoagland’s solution depleted in phosphate was used to water the plants. Several studies have shown that similar conditions allow both crop species to grow and the establishment of an efficient AMF symbiosis (Gutjahr et al., 2008; Fiorilli et al., 2018; Campo and San Segundo, 2020; Campo et al., 2020; Guo et al., 2022).

### *Japonica* cultivars respond better to mycorrhization than *indica* cultivars

Under our growing conditions, *japonica* rice appeared to be more intensely colonised than *indica* rice. (**Figure 1**; **Supplementary Table 2**). The result of upland *japonica* rice cultivars being more colonised and responding more to AMF colonisation than flooded *indica* rice cultivars is shared by several studies, considering their different root architecture and the co-evolution between AMF and upland rice in aerobic selective condition, optimizing their interaction (Diedhiou et al., 2016; Davidson et al., 2019). It is important to note that we selected two *indica* rice cultivars compared to four *japonica*, depending on the valuable factors listed in **Table 1**. This study should be extended to more rice genotypes from both subspecies to understand if this pattern can be generalised. The global understanding of how rice mycorrhization is affected by host genotype is currently limited. In a published report, the mycorrhizal growth response (MGR, corresponding to the ratio between the dry weight of mycorrhized and control plants) was measured for 64 rice genotypes from different subspecies 4 weeks after inoculation with *F. mosseae*. Differences between genotypes were observed but not related to *indica* or *japonica* origin (Suzuki et al., 2015). A possible explanation is that variations in AMF recognition receptors between host genotypes affect their symbiotic compatibility. OsCERK1, a LysM receptor-like kinase that is essential for AMF recognition and activation of the symbiosis pathway, is also responsible for root branching in rice (Chiu et al., 2018; Choi et al., 2018). It has been proposed that natural variation between allelic variants of the *OsCERK1* gene in different rice cultivars may affect their symbiotic compatibility with *R. irregularis* DAOM 197198 (Huang et al., 2020). A higher level of AMF colonisation, 14 days post inoculation, was reported for eleven *indica* rice cultivars, compared to eight *japonica*. The difference was proposed to be related to an underrepresented *OsCERK1* haplotype, absent in *japonica* rice and present in their selected *indica* cultivars (Lefebvre, 2020). In our study, we obtained opposite results, but we chose to harvest the rice after two months of growth, which, in addition to the different substrate used, may explain the difference. Several reports have shown that root colonisation of Nipponbare becomes clearly visible after 14 days post inoculation, and arbuscules after 3 to 4 weeks (Gutjahr et al., 2015; Guo et al., 2022; Liu et al., 2022). It would be interesting to assess whether rice cultivar also affects the kinetics of AMF establishment and functioning in the long term.

### Beneficial effects on resistance to Xoo are highly dependent on rice cultivar and AMF genotypes

As plant colonisation by AMF has often been shown to be associated with a better protection against pathogens, we conducted biocontrol trials against Xoo. We observed a general tendency for symptoms to decrease, which was statistically significant for some AMF-rice cultivar combinations (**Figure 4**; **Supplementary Table 2**). Only two among 18 showed an increase in the leaf symptoms when associated with an AMF (Phka Rumduol and Azucena associated with RIN). Almost all well-colonised *japonica* rice species showed a significant reduction of at least one Xoo-induced symptoms thanks to MIR, particularly with Zhonghua 11 and Nipponbare cultivars. The most-colonised rice cultivar, Nipponbare, see chlorosis and necrosis symptoms reduced in both FM and RIN conditions. With the most compatible one, this reduction of symptoms can be noticed but is not statistically significant. This reduction of symptoms becomes statistically significant in a repeated study on 20 non-mycorrhizal Nipponbare and 20 associated with RIR (**Additionnal file 6**: **Supplementary Figure 3**). An induction of 39%, 107% and 187% of Nipponbare’s maximum height, shoot and root dry weight, respectively was shown, linked with a reduction of chlorosis symptoms by 32% at 14 days post-clipping. It is noteworthy that IR64, a rice variety three times less intensively mycorrhized, also shows reduced chlorosis symptoms when mycorrhized with FM or RIR. A possible explanation could reside in the differences between rice genotypes themselves, being more or less sensitive to AMF colonisation and their established interaction, having then important or little to no effects on rice growth phenotype, but notable impacts on rice nutrient physiology and defence responses. Our results highlight here that symbiotic compatibility between AMF and rice species states for a sufficient amount of colonisation allowing significant nutrient starvation response inhibition and phytopathogen-induced symptoms reduction.

The potential of biological control by AMF symbiosis is well documented in the literature, although pathogen-dependent. Mycorrhization of *japonica* rice cultivars by AMF genotypes such as *R. intraradices, R. irregularis* or *F. mosseae* showed both a local and a systemic defence response against *Pyricularia oryzae*, sometimes associated with an increase in shoot biomass (Campos-Soriano et al., 2010 ; Campos-Soriano et al., 2012; Campo et al., 2020). In *Triticum aestivum* cv. Chinese Spring associated with *F. mosseae*, the AMF symbiosis confers both a positive effect on growth and resistance to *X. translucens* (Fiorilli et al., 2018). Two American rice cultivars inoculated with a mixture of AMF genotypes showed increased susceptibility to two insects and *Rhizoctonia solani* infections, but without growth defects or nutrient losses, suggesting an effect of symbiosis on defence vs growth trade-off (Bernaola et al., 2018b).

### *Oryza sativa* cv *japonica* Nipponbare is the best AMF-responsive rice cultivar

Many research studies focus on the association between *R. irregularis* and Nipponbare (Gutjahr et al., 2009; Gutjahr et al., 2015; Campo et al., 2020; Guo et al., 2022; Liu et al., 2022). Since they are both models (sequenced genomes, easy to grow, transform and store, controlled lifecycle), deepening our knowledge of their interaction at different life stages of both organisms may shed light on how rice interacts with and benefits from AMF and *vice-versa*. Under our conditions, Nipponbare was the rice cultivar with the best level of mycorrhization, regardless of the AMF inoculated (**Figure 1**; **Supplementary Table 2**). There is no significant negative effect of mycorrhization on its growth, with a tendency of AMF symbiosis to increase both height and weight (**Figure 3**). After Xoo infection, a significant reduction of symptoms was observed with FM and RIN (**Figure 4**). To further confirm this, the experiment was repeated (n = 20) for Nipponbare in combination with *R. irregularis* DAOM197198 and showed then a clear positive effect on growth promotion (height, root and leaf weight) and on biocontrol against Xoo (**Supplementary Figure 5**). At the molecular level, RT-qPCR analysis showed that the expression of cellular development marker genes was induced by mycorrhization, even after two months of growth (**Figure 5**; **Supplementary Table 3**). Under these conditions, the overall expression of nutrient transporter and defence genes was down-regulated. As these genes are known to be induced upon starvation, Nipponbare in symbiosis with AMF could then be considered to be in a healthy state.

Our results are in agreement with previous work. On Nipponbare, *Glomus intraradices*, which could be classified as *R. irregularis* according to the current AMF phylogeny, colonises more than 60% of the large lateral roots and increases both dry weight and coronary root length (Gutjahr et al., 2009). Colonisation kinetics of *R. irregularis* on this cultivar showed high colonisation rates (up to 87% of root length, of which 59% contained arbuscules) and the presence of viable arbuscules and vesicles up to six weeks after inoculation (Gutjahr et al., 2015; Liu et al., 2022). Here we show that even after ten weeks, arbuscules and vesicles were still clearly visible and in larger numbers. A study on the effect of pre-inoculation of *F. mosseae* on Nipponbare before transplanting in the field showed a root colonisation rate of 65%, an arbuscule content of 1.3% and a vesicle content of 6% (Sisaphaithong et al., 2017), consistent with our results. Growing conditions and practices may have an impact on this low arbuscule content. After 10 weeks of association with *F. mosseae*, eight temperate *japonica* cultivars used in Spanish or Italian fields had between 20 and 60% of arbuscules in their mycorrhized roots, with non-significant to beneficial effects on both rice growth and *P. oryzae* tolerance (Campo et al., 2020). The geographical origin and genotype of the host may determine its symbiotic compatibility with different AMF genotypes.

### Defence potential imprint revealed by systemic molecular analyses

The description of the phenotypic responses (rice growth and tolerance to Xoo) of different rice cultivars to inoculation with each AMF genotype allowed us to assess the symbiotic compatibility between them. To understand how AMF symbiosis affects the molecular functioning in two rice cultivars with contrasting phenotypic responses to AMF, the leaf molecular responses of Nipponbare and IR64 after six weeks of interaction were studied. Local molecular responses to the establishment of the mycorrhization have been well studied in rice roots (Güimil et al., 2005; Gutjahr et al., 2009; Campos-Soriano et al., 2010, reviewed in Choi et al., 2018), but the systemic effects on the responses of rice leaves are still poorly described. A panel of rice marker genes involved in development, nutrient status, phytohormone signalling and defence responses (listed in **Table 2**, detailed in the results) was selected, and their expression analysed in healthy 50-day old leaves of Nipponbare and IR64.

First of all, the response of Nipponbare leaves to root mycorrhization was consistent with our phenotypic results. We observed a modulation of developmental gene expression, coupled with a reduced starvation response (repression of Pi starvation’s marker genes and a nitrate reductase marker gene, **Figure 5**; **Supplementary Table 3**). Defence related genes expression was not significantly to negatively affected by AMF symbiosis in Nipponbare, suggesting that mycorrhization does not induce a systemic defence response under healthy conditions. Overall, mycorrhization of the *japonica* rice Nipponbare highlights an improvement of rice’s development and nutritional status, to be linked with the reported increase in growth in this most intensely mycorrhized rice. This imprint on rice’s expression pattern hints for a better overall state and tolerance against abiotic as well as biotic stressors. The study of the effect of either one of them on mycorrhized Nipponbare is interesting to assess which specific systemic mechanisms will act on the trade-off between growth and tolerance.

Mycorrhized IR64 plants showed a different response to AMF symbiosis. A non-significant repression of developmental and nutrient starvation response marker genes was observed, coupled with a non-significant induction of the defence response (**Figure 5**; **Supplementary Table 3**). Under our growth conditions, mycorrhization of IR64 does not seem to be as beneficial as of Nipponbare and this is still to be linked with phenotypic results (i.e. a non-significant to negative impact of mycorrhization on IR64’s growth with a less mycorrhized cultivar).

The nutritional status of both our rice cultivar were investigated thanks to iron, phosphate and nitrate-related genes expression. There is a strong trend of induction of *OsIRO2* expression in leaves of Nipponbare interacting with RIN (**Figure 5**; **Supplementary Table 3**; **Supplementary Figure 3**). This transcription factor is induced under Fe deficiency, modulating key genes involved in iron uptake in rice (*OsNAS1*, *OsTOM1* or *OsYSL15*), with *IRO2*-overexpressing rice showing improved Fe-deficiency tolerance compared to the non-transgenic lines (Ogo et al., 2006; Ogo et al., 2011). Mycorrhization may affect iron homeostasis, *via* its effect on *IRO2* expression, affecting Fe uptake and translocation to shoots and grains (Ogo et al., 2011) but little research has been done on this subject. Leaves of the *japonica* rice Senia show an increase of *IRO2* expression during mycorrhization with RIR, while wheat symbiosis with FM triggers an accumulation of Fe-uptake related proteins in roots, linked with a translocation of iron from roots to shoots, suggesting both a beneficial effect of mycorrhization on iron homeostasis in mycorrhized hosts (Campos-Soriano et al., 2012; Fiorilli et al., 2018).

AMF have been demonstrated to increase the bioavailability of essential nutrients such as nitrogen and phosphate to their host roots. This capacity can be linked with the repression of *OsNIA1* and Pi-starvation-related genes expression in the leaves of our two studied rice cultivars. (**Figure 5**; **Supplementary Table 3**; **Supplementary Figures 3**, **4**). These results are in line with previous reports, and specifically results showing the repression of *OsSPX3, OsPAP23* and *OsMGD2* expression in Loto rice leaves in association with FM (Liu et al., 2007; Gutjahr et al., 2008; Fiorilli et al., 2018; Campo and San Segundo, 2020; Vannini et al., 2021).

The expression of marker genes for phytohormones biosynthesis or signalling in leaves was not significantly affected by AMF symbiosis, except for a repression of the SA perception gene (*OsNPR1*) in leaves of IR64 (**Figure 5**; **Supplementary Table 3**; **Supplementary Figure 4**). Recent literature reports contradictory results on the effect of mycorrhization on SA pathways in wheat or rice: the first is not affected on any SA-related pathways during FM mycorrhization, while *OsNPR1* is induced in some *japonica* rice cultivars (Campos-Soriano et al., 2012; Fiorilli et al., 2018; Tian et al., 2019). Mycorrhization effect on SA-related pathways may be plant species-dependent (Campo and San Segundo, 2020).

Under our conditions, ethylene and jasmonate related genes were not significantly repressed in both cultivars (**Figure 5**; **Supplementary Table 3**). Ethylene biosynthesis is known to be induced in tomato and wheat and repressed in rice leaves under mycorrhization, not significantly in our case (Fiorilli et al., 2018; Campo and San Segundo, 2020). Jasmonate-related genes are previously reported to be modulated by mycorrhization in different rice cultivars, both on its biosynthesis or signalling (Campos-Soriano et al., 2012; Campo and San Segundo, 2020). MIR is reported to occur via JA-related pathways, leading to reduced symptoms and pathogen load in multiple host-pathogen interactions (Dowarah et al., 2021). The molecular responses to Xoo infection may allow us to understand if the reduction of symptoms occurring in both our rice cultivars are linked with MIR, a primed induction of defence responses, responding faster and more efficiently than non-mycorrhized controls, or by an overall better rice health state (related to both growth and nutritional status improvement). If the MIR hypothesis appears to be true, deeper transcriptional analyses are to be conducted to understand by which mechanisms MIR occurs.

Overall, the systemic response of rice to AMF symbiosis is dependent on rice cultivar and AMF genotype but can be linked to an overall improvement in rice health.

## Conclusion

In this study we showed that the establishment of the AMF symbiosis and its effects on rice depends on both the rice cultivar and the AMF genotype for each variable studied. We found that mycorrhizal growth enhancement and induced resistance to Xoo strongly depends on both rice variety and AMF genotype. In our study and under our conditions, *japonica* rice chosen subspecies tend to be more colonised and have more benefits on growth and defence responses than *indica* ones. In the model rice cultivars Nipponbare and IR64, root colonisation rate, growth enhancement or shortage in both shoot and roots can be associated with a systemic modification of molecular pathways in leaves. These differences in rice response raise the question of how beneficial the AMF symbioses really are. In some cases, AMF interactions are detrimental to the growth of the plant host or its response to the environment (Jin et al., 2017; Bernaola et al., 2018b). The assumption that AMF symbiosis can be viewed as an equilibrium between mutualism and parasitism, with symbiont considered as more or less efficient and cooperative partners (Kiers et al., 2011; Kaur et al., 2022), may be closer to biological reality. In our study, we have identified rice-AMF combinations that are able to develop into a functional symbiosis with positive effects on both rice growth and tolerance to phytopathogens. These combinations should now be tested in unflooded rice field conditions with low Pi to unravel their true potential.

## Data availability statement

The original contributions presented in the study are included in the article/**Supplementary Material**. Further inquiries can be directed to the corresponding author.

## Author contributions

LG: Conceptualization, Data curation, Formal Analysis, Investigation, Methodology, Visualization, Writing – original draft. LJ: Formal Analysis, Investigation, Methodology, Writing – original draft. NB: Investigation, Methodology, Writing – original draft. LM: Conceptualization, Funding acquisition, Project administration, Supervision, Writing – review & editing. PC: Conceptualization, Funding acquisition, Project administration, Resources, Supervision, Validation, Writing – review & editing.
