# Supplementary material for: Symbiotic compatibility between rice cultivars and arbuscular mycorrhizal fungi genotypes affects rice growth and mycorrhiza-induced resistance
Source: Front Plant Sci. 2023 Oct 24;14:1278990. doi: 10.3389/fpls.2023.1278990 (PMC10628536; doi:10.3389/fpls.2023.1278990)
Supplement: Supplementary file 1 [file DataSheet_1.pdf]

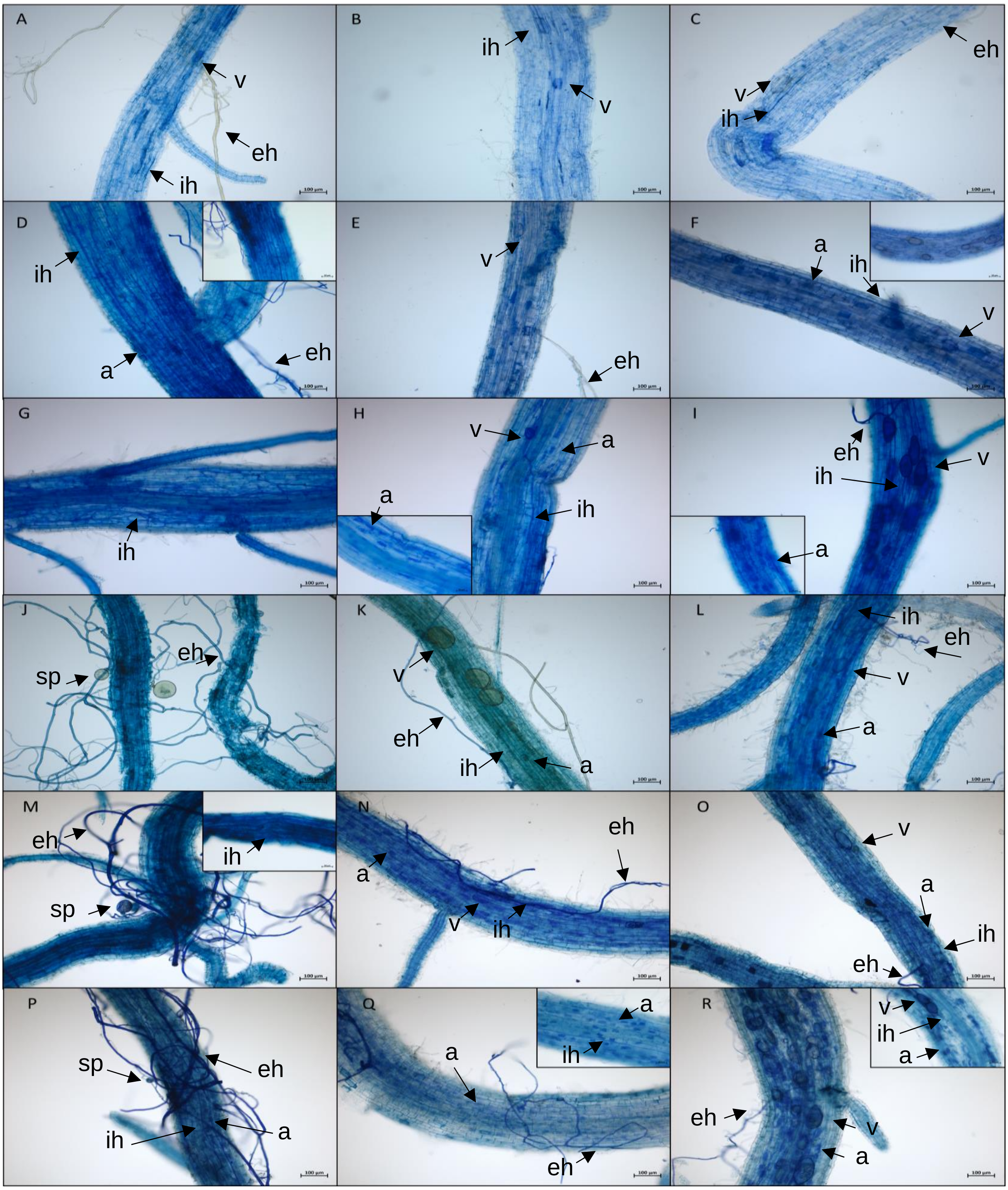

Supplementary Figure 1. Colonisation of each rice genotype root with each AMF species.

A, D, G, J, M, P= Roots colonised with *Funneliformis mosseae*. B, E, H, K, N, Q= Roots colonised with *Rhizophagus intraradices.* C, F, I, L, O, R= Roots colonised with *Rhizophagus irregularis*. A, B, C= IR64 roots. D, E, F= Phka Rumduol roots. G, H, I= Azucena roots. J, K, L= Kitaake roots. M, N, O= Nipponbare roots. P, Q, R= Zhonghua 11 roots. Eh= external hyphae. Ih= internal hyphae. V= vesicules. A= arbuscules. Sp= spores.
