# Supplementary material for: Symbiotic compatibility between rice cultivars and arbuscular mycorrhizal fungi genotypes affects rice growth and mycorrhiza-induced resistance
Source: Front Plant Sci. 2023 Oct 24;14:1278990. doi: 10.3389/fpls.2023.1278990 (PMC10628536; doi:10.3389/fpls.2023.1278990)

Supplementary Figure 2. Developmental stage of each rice genotype with each AMF species. Grey = *indica* rice species. Blue = *japonica* rice species. Green, yellow, red and blue stickers indicate non-inoculated control (CT), *Rhizophagus irregularis* (RIR), *R. intraradices* (RIN) or *Funneliformis mosseae* (FM) associated-plants.

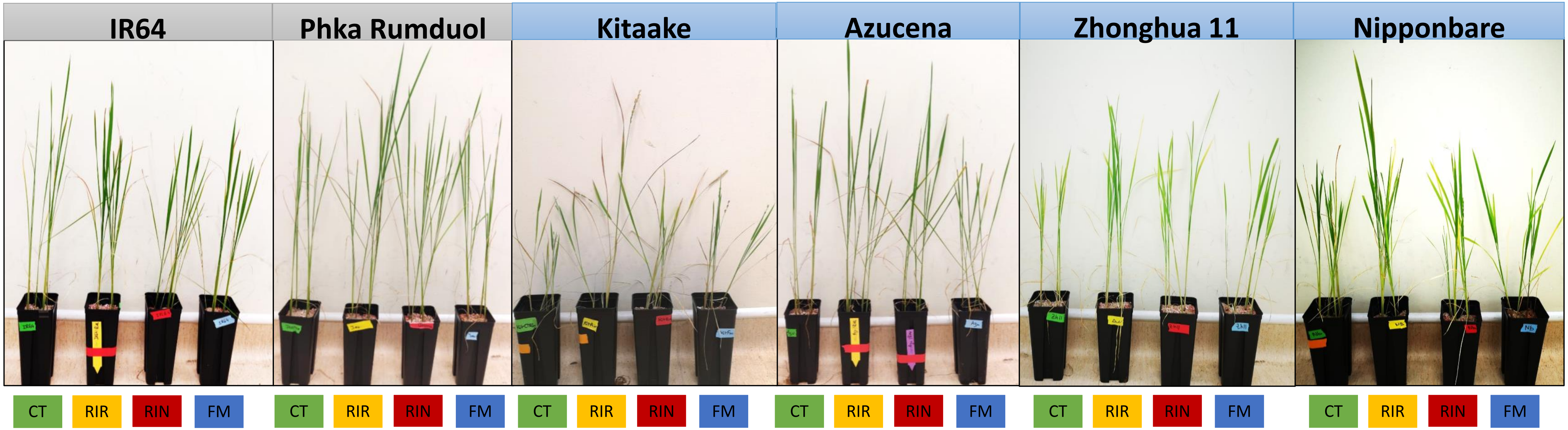

Supplement: Supplementary file 2 [file DataSheet_2.pdf]
