# Supplementary material for: Symbiotic compatibility between rice cultivars and arbuscular mycorrhizal fungi genotypes affects rice growth and mycorrhiza-induced resistance
Source: Front Plant Sci. 2023 Oct 24;14:1278990. doi: 10.3389/fpls.2023.1278990 (PMC10628536; doi:10.3389/fpls.2023.1278990)
Supplement: Supplementary file 4 [file DataSheet_4.pdf]

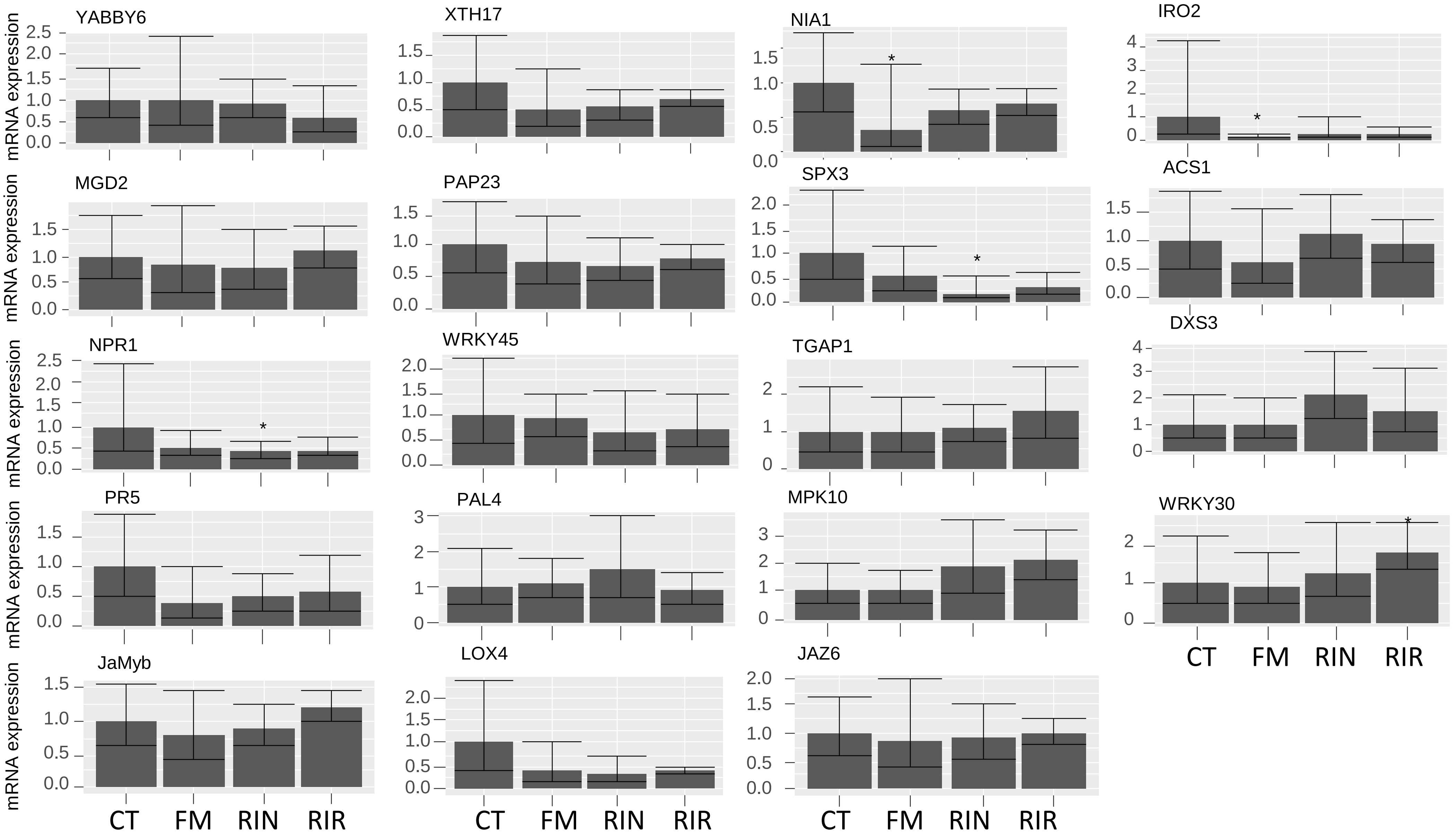

Supplementary Figure 4. Relative expression of marker genes of development, hormonal balances, nutrition and defence in IR64 leaves in association with no AMF species, *Funneliformis mosseae* (FM), *Rhizophagus intraradices* (RIN) or *R. irregularis* (RIR). N= 4. “*”= statistically different from the control group, p-value < 0.05
