# Supplementary material for: Symbiotic compatibility between rice cultivars and arbuscular mycorrhizal fungi genotypes affects rice growth and mycorrhiza-induced resistance
Source: Front Plant Sci. 2023 Oct 24;14:1278990. doi: 10.3389/fpls.2023.1278990 (PMC10628536; doi:10.3389/fpls.2023.1278990)
Supplement: Supplementary file 5 [file DataSheet_5.pdf]

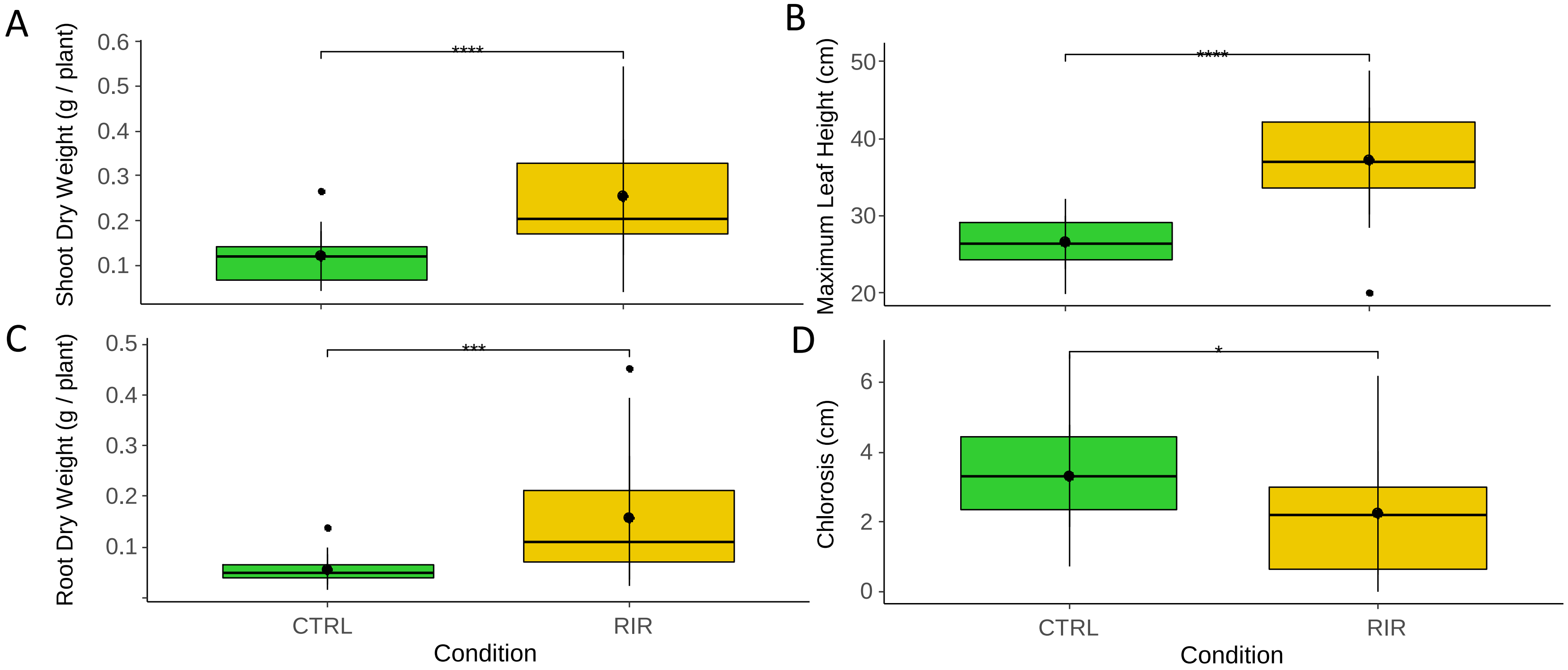

Supplementary Figure 5. Nipponbare's phenotypes when interacting with *Rhizophagus irregularis*. A: Shoot dry weight (g / plant). B: Maximum height of rice plants (cm). C: Root dry weight (g / plant). D: Size of chlorosis symptoms (cm), depending on AMF inoculation (green = no AMF, yellow = RIR). *: p value < 0.05; ****: p value < 0.001.
