# Supplementary material for: Symbiotic compatibility between rice cultivars and arbuscular mycorrhizal fungi genotypes affects rice growth and mycorrhiza-induced resistance
Source: Front Plant Sci. 2023 Oct 24;14:1278990. doi: 10.3389/fpls.2023.1278990 (PMC10628536; doi:10.3389/fpls.2023.1278990)
Supplement: Supplementary file 6 [file Table_1.docx]

**Supplementary Table 1.** **Plant Nutritional solution composition.** Adapted from Hoagland & Anon, 1950 and Campo & San Segundo, 2020.

| **Solution Name** | **Salt** | **Final concentration** |
| --- | --- | --- |
| **(NH_4_)** | (NH_4_)_2_SO_4_ | 0,25 mM |
| **MgSO_4_** | MgSO_4_.7H_2_O | 1 mM |
| **Ca(NO_3_)_2_** | Ca(NO_3_)_2_.4H_2_O | 2,5 mM |
| **KNO_3_** | KNO_3_ | 2,5 mM |
| **Oligoelements** | MnSO_4_.H_2_O | 8 µM |
|  | (NH_4_)_6_Mo_7_O_24_.4H_2_O | 0,14 µM |
|  | ZnSO_4_.7H_2_O | 0,24 µM |
|  | CuSO_4_.5H_2_O | 0,26 µM |
|  | H_3_BO_3_ | 24 µM |
| **Iron (sequestrene)** | Fer-EDDHA | 44 µM |
| **KH_2_PO_4_** | KH_2_PO_4_ | 25 µM |
