# Supplementary material for: Symbiotic compatibility between rice cultivars and arbuscular mycorrhizal fungi genotypes affects rice growth and mycorrhiza-induced resistance
Source: Front Plant Sci. 2023 Oct 24;14:1278990. doi: 10.3389/fpls.2023.1278990 (PMC10628536; doi:10.3389/fpls.2023.1278990)
Supplement: Supplementary file 7 [file Table_2.docx]

**Supplementary Table 2. Summary of the impact of each AMF on each rice genotype on phenotypic and pathogen tolerance traits**. n = 5 for the mycorrhizal index (global intensity of mycorrhization, arbuscular content, %); n= 20 for biomass and symptoms analyses. Absolute mean values and relative mean compared with the control ($\frac{Treatment-Control}{Control} \times100$).

| **Rice cultivar** | **AMF species** | **Colonisation rate (%)** | **Arbuscular content (%)** | **Maximum Height (cm)** | **Relative Height (%)** | **Shot Fresh Weight (g)** | **Relative Shoot Fresh Weight (%)** | | **Roots**  **Fresh Weight (g)** | **Relative Root**  **Fresh Weight (%)** | **Shoot**  **Dry Weight (g)** | **Relative Shoot**  **Dry Weight (%)** | **Chlorosis Lesions (cm)** | **Relative Chlorosis Spread (%)** | **Necrosis Lesions (cm)** | **Relative Necrosis Spread (%)** |
| --- | --- | --- | --- | --- | --- | --- | --- | --- | --- | --- | --- | --- | --- | --- | --- | --- |
| **IR64** | **CT** | - | - | 43.57 |  | 0.49 |  | 0.25 | |  | 0.14 |  | 8.78 |  | 0.77 |  |
|  | **FM** | 28.80 | 3.48 | 35.37 | -18.81 | 0.40 | -19.06 | 0.23 | | -6.53 | 0.12 | -14.58 | 6.67 | -24.04 | 1.09 | 41.98 |
|  | **RIN** | 46.80 | 13.80 | 37.42 | -14.12 | 0.39 | -19.67 | 0.25 | | 0.00 | 0.12 | -18.06 | 7.37 | -16.07 | 1.16 | 51.37 |
|  | **RIR** | 33.00 | 7.73 | 42.54 | -2.38 | 0.56 | 13.73 | 0.34 | | 37.96 | 0.18 | 27.78 | 6.42 | -26.89 | 0.83 | 8.47 |
| **Phka Rumduol** | **CT** | - | - | 47.27 |  | 0.43 |  | 0.20 | |  | 0.13 |  | 6.27 |  | 0.57 |  |
|  | **FM** | 27.70 | 3.58 | 46.10 | -2.49 | 0.41 | -4.42 | 0.22 | | 13.33 | 0.12 | -9.02 | 5.60 | -10.74 | 1.07 | 86.76 |
|  | **RIN** | 27.20 | 6.08 | 52.23 | 10.49 | 0.49 | 12.79 | 0.24 | | 23.08 | 0.15 | 12.03 | 6.26 | -0.18 | 1.18 | 106.27 |
|  | **RIR** | 27.80 | 6.08 | 57.15 | 20.89 | 0.69 | 59.30 | 0.49 | | 148.72 | 0.20 | 48.87 | 5.28 | -15.78 | 0.96 | 67.77 |
| **Kitaake** | **CT** | - | - | 24.65 |  | 0.19 |  | 0.11 | |  | 0.09 |  | 3.58 |  | 1.37 |  |
|  | **FM** | 43.20 | 3.92 | 29.63 | 20.18 | 0.50 | 163.16 | 0.30 | | 177.78 | 0.21 | 125.81 | 3.54 | -0.90 | 1.06 | -22.79 |
|  | **RIN** | 68.50 | 20.50 | 33.75 | 36.92 | 0.68 | 259.47 | 0.40 | | 270.37 | 0.30 | 221.51 | 5.32 | 48.81 | 1.18 | -13.81 |
|  | **RIR** | 70.90 | 35.90 | 33.38 | 35.40 | 0.54 | 181.58 | 0.32 | | 196.30 | 0.26 | 178.49 | 4.11 | 15.05 | 0.80 | -41.56 |
| **Azucena** | **CT** | - | - | 49.34 |  | 0.63 |  | 0.75 | |  | 0.15 |  | 3.06 |  | 0.29 |  |
|  | **FM** | 60.90 | 3.22 | 39.76 | -19.42 | 0.43 | -31.52 | 0.43 | | -41.88 | 0.12 | -21.77 | 2.02 | -34.01 | 0.23 | -20.75 |
|  | **RIN** | 61.50 | 22.30 | 48.19 | -2.34 | 0.57 | -8.80 | 0.46 | | -38.26 | 0.15 | 3.40 | 5.16 | 68.61 | 0.47 | 58.84 |
|  | **RIR** | 73.30 | 38.50 | 52.01 | 5.40 | 0.79 | 26.24 | 0.83 | | 11.14 | 0.21 | 40.14 | 4.40 | 43.74 | 0.89 | 202.04 |
| **Zhongua 11** | **CT** | - | - | 33.42 |  | 0.32 |  | 0.27 | |  | 0.12 |  | 9.48 |  | 2.10 |  |
|  | **FM** | 72.80 | 5.24 | 34.96 | 4.62 | 0.40 | 26.03 | 0.25 | | -9.26 | 0.14 | 13.93 | 5.27 | -44.49 | 0.74 | -65.00 |
|  | **RIN** | 63.90 | 24.20 | 39.08 | 16.94 | 0.53 | 66.67 | 0.30 | | 9.26 | 0.18 | 49.18 | 6.80 | -28.35 | 0.70 | -66.67 |
|  | **RIR** | 70.90 | 31.40 | 39.94 | 19.51 | 0.64 | 103.17 | 0.45 | | 65.56 | 0.22 | 80.33 | 6.74 | -28.91 | 0.75 | -64.43 |
| **Nipponbare** | **CT** | - | - | 30.41 |  | 0.38 |  | 0.36 | |  | 0.15 |  | 8.38 |  | 1.80 |  |
|  | **FM** | 79.00 | 5.00 | 31.58 | 3.84 | 0.41 | 8.16 | 0.26 | | -27.89 | 0.15 | 4.08 | 5.04 | -39.81 | 0.39 | -78.11 |
|  | **RIN** | 77.90 | 41.20 | 32.19 | 5.85 | 0.39 | 2.63 | 0.19 | | -47.89 | 0.15 | 0.00 | 5.53 | -33.95 | 0.23 | -87.44 |
|  | **RIR** | 83.90 | 49.40 | 35.73 | 17.49 | 0.60 | 57.89 | 0.42 | | 17.75 | 0.22 | 51.70 | 7.87 | -5.98 | 0.64 | -64.61 |
