# Supplementary material for: Symbiotic compatibility between rice cultivars and arbuscular mycorrhizal fungi genotypes affects rice growth and mycorrhiza-induced resistance
Source: Front Plant Sci. 2023 Oct 24;14:1278990. doi: 10.3389/fpls.2023.1278990 (PMC10628536; doi:10.3389/fpls.2023.1278990)
Supplement: Supplementary file 8 [file Table_3.docx]

**Supplementary Table 3**. Statistical comparison of marker genes expression of development, hormonal balances, nutrition and defence in mycorrhized rice leaves, compared with control, non-mycorrhized ones ($\Delta Ct,$linear regression, significant when p-value < 0,05. Bold numbers: statistically significant. Italic number: tendencies (p-value < 0,10)).
